# Robust Multiple Importance Sampling with Tsallis *φ*-Divergences

**DOI:** 10.3390/e24091240

**Published:** 2022-09-03

**Authors:** Mateu Sbert, László Szirmay-Kalos

**Affiliations:** 1 Institute of Informatics and Applications, University of Girona, 17071 Girona, Spain; 2 Department of Control Engineering and Information Technology, Budapest University of Technology and Economics, 1111 Budapest, Hungary

**Keywords:** multiple importance sampling, Monte Carlo integration, φ-divergence, f-divergence, Tsallis divergence, Kullback–Leibler divergence, chi-square divergence, image synthesis

## Abstract

Multiple Importance Sampling (MIS) combines the probability density functions (pdf) of several sampling techniques. The combination weights depend on the proportion of samples used for the particular techniques. Weights can be found by optimization of the variance, but this approach is costly and numerically unstable. We show in this paper that MIS can be represented as a divergence problem between the integrand and the pdf, which leads to simpler computations and more robust solutions. The proposed idea is validated with 1D numerical examples and with the illumination problem of computer graphics.

## 1. Introduction and Previous Work

Multiple Importance Sampling (MIS) [1,2] has been proven efficient in Monte Carlo integration. It is able to preserve the advantages of the combined techniques and requires only the calculation of the pdfs of all methods when a sample is generated with one particular method. The weighting scheme applied in MIS depends on the pdfs of the individual techniques and also on the number of samples generated with each of them.

MIS has been applied in a number of rendering algorithms, and its variance is extensively studied [1]. Several estimators have been proposed that are better than balance heuristics with equal sample budgets [3,4,5,6]. Lu et al. [7] proposed an adaptive algorithm for environment map illumination, which used the Taylor series approximation of the variance around an equal sample budget case. In [8,9], different equal sample number strategies were analyzed. Sbert et al. [10] considered the cost associated with the sampling strategies and obtained an adaptive solution by optimizing the variance using the Newton–Raphson method [11]. In [12], the Kullback–Leibler divergence was optimized instead of the variance. Several authors have shown that the variance of an importance sampling estimator is equal to a chi-square divergence [13,14,15,16], which, in turn, can be approximated by the Kullback–Leibler divergence up to the second order [17]. The optimal sample budget has also been targeted with neural networks [15,18]. Recently, a theoretical formula has been elaborated for the weighting functions [19]. In [16], the balance heuristic estimator was generalized by decoupling the weights from the sampling rates, and implicit solutions for the optimal case were given.

These techniques offer lower variance and, therefore, theoretically outperform MIS with equal number of samples. However, equations determining the optimal weighting and sample budget require the knowledge of the integrand and numerical solution methods. In computer graphics, for example, this integrand is not analytically available, so previous discrete samples should be used for the approximation, which introduces errors in the computation. Thus, it is not guaranteed that a theoretically superior estimator also performs better in practice.

This paper proposes an adaptive approach to automatically determine the sampling budgets of the combined methods based on the statistics of previous samples. To improve the robustness, instead of directly optimizing the variance, we make the combined pdf mimic the integrand by minimizing the divergence between them. From the possible alternatives, the Tsallis φ-divergence is used since this leads to a general and stable approach. We show that finding the optimum in all cases, including also Hellinger, chi-square, Kullbach–Leibler divergences, and the variance, boils down to a non-linear equation stating that the γ-moments of the different combined techniques must be equal. This equation has unique root that can be found by Newton–Raphson iteration.

The organization of the paper is the following. In Section 2, the multi-sample version of the MIS estimator is reviewed where the number of samples allocated to each combined technique is fixed deterministically. Section 3 summarizes the one-sample MIS estimator where the particular sampling method is also selected randomly. In Section 4, the adaptation of the MIS weighting functions is discussed and we argue that the adaptation should be controlled not by the estimated variance but by an appropriately chosen divergence. Section 5 reformulates the MIS problem as the optimization of divergences and introduces the γ-moments. In Section 6, the details of the numerical optimization are discussed. Finally, the results are demonstrated with numerical examples and the direct lighting solution of computer graphics.

## 2. Multi-Sample Balance Heuristic Mis

Suppose we wish to estimate the value of integral ∫f(x)dx and have *m* proposal pdfs $p_{i}\left(x\right)$ to generate the *j*th sample $X_{ij}$ of method *i* in the domain of the integral. With method *i*, $n_{i}$ independent samples are drawn, so the total number of samples is $\sum_{i=1}^{m}n_{i}=N$. When the sample numbers $n_{i}$ are fixed deterministically, the approach is called *multi-sample*.

The multi-sample MIS estimator [1] has the following expression:

(1) $$F=\sum_{i=1}^{m}\frac{1}{n_{i}}\sum_{j=1}^{n_{i}}w_{i}\left(X_{ij}\right)\frac{f(X_{ij})}{p_{i}\left(X_{ij}\right)},$$

where the weights satisfy the normalization condition:

$$\sum_{i=1}^{m}w_{i}\left(x\right)=1.$$

Integral estimator *F* is unbiased, as its expected value μ is equal to the integral:

(2) $$\mu =E\left[F\right]=\sum_{i=1}^{m}\frac{1}{n_{i}}\sum_{j=1}^{n_{i}}\int \frac{w_{i}\left(x\right)f\left(x\right)}{p_{i}\left(x\right)}p_{i}\left(x\right)dx=\sum_{i=1}^{m}\mu_{i}=\int f\left(x\right)dx$$

where $\mu_{i}=\int w_{i}\left(x\right)f\left(x\right)dx$. The variance of the integral estimator is

(3) $$V\left[F\right]=\sum_{i=1}^{m}\int \frac{w_{i}^{2}\left(x\right)f^{2}\left(x\right)}{n_{i}p_{i}\left(x\right)}dx-\sum_{i=1}^{m}\frac{\mu_{i}^{2}}{n_{i}}.$$

The variance of the estimator depends on the combination weights $w_{i}\left(x\right)$. The first step of their definition is to propose an algebraic form. The *balance heuristic* states that the weight of method *i* at domain point *x* should be proportional to the density of samples generated by method *i* in this point:

(4) $$w_{i}\left(x\right)=\frac{\alpha_{i}p_{i}\left(x\right)}{p(\alpha ,x)}$$

where $\alpha_{i}=n_{i}/N$ is the *fraction of the samples* allocated to method *i*, and p(α,x) is the *mixture pdf*:

(5) $$p\left(\alpha ,x\right)=\sum_{k=1}^{m}\alpha_{k}p_{k}\left(x\right).$$

Substituting this weighting function into the MIS estimator formulas, we obtain the multi-sample balance heuristics estimator:

(6) $$F=\frac{1}{N}\sum_{i=1}^{m}\sum_{j=1}^{n_{i}}\frac{f(X_{i,j})}{p(\alpha ,X_{i,j})}.$$

The variance of the balance heuristics estimator is

(7) $$V\left[F\right]=\frac{1}{N}\left(\int \frac{f^{2}\left(x\right)}{p(\alpha ,x)}dx-\sum_{i=1}^{m}\alpha_{i}{\mu^{\prime }}_{i}^{2}\right),$$

where ${\mu^{\prime }}_{i}=\mu_{i}/\alpha_{i}$.

## 3. One-Sample Balance Heuristic MIS

In the *one-sample* version of MIS, the numbers of samples used by particular techniques are not determined before starting the sampling process, but for each sample, the sampling method itself is selected randomly with the probability of fraction parameter $\alpha_{i}$. As this approach introduces additional randomization, its variance is larger than that of the multi-sample version, but can also be used in cases when the number of total samples is less than the number of combined methods.

The one-sample balance heuristic estimator is given by

(8) $$F=\frac{f(X)}{p(\alpha ,X)}$$

with variance

(9) $$V\left[F\right]=\int \frac{f^{2}\left(x\right)}{p(\alpha ,x)}dx-\mu^{2}.$$

## 4. Adaptive MIS

Having the algebraic form of the weight function, the task is to find the optimal fractions $\alpha_{i}$ with the constraint that the sum of sample numbers must be equal to the total sample budget, i.e., $\sum_{i=1}^{m}\alpha_{i}=1$. One possibility is to use some heuristics before starting the sampling process. A simple example is the *equal sample count MIS* that sets all fractions to be equal.

Adaptive techniques, on the other hand, try to minimize the error by controlling the fractions on-the-fly as new samples introduce additional information. During this, the following issues need to be considered:

- The integrals in the variance cannot be computed analytically, but must be estimated from the available finite number of samples drawn from the sampling distribution. This uncertainty may significantly affect the goodness of the final results.
- The optimization process should be fast and should not introduce too high overhead.

Unfortunately, the direct optimization for the variance does not meet these requirements. The integral of the variance can be estimated from the available samples $X_{i}$ drawn from mixture distribution p(α,x) as:

(10) $$\int \frac{f^{2}\left(x\right)}{p(\alpha ,x)}dx=E_{p}\left[\frac{f^{2}\left(x\right)}{p^{2}\left(\alpha ,x\right)}\right]\approx \frac{1}{N}\sum_{i=1}^{N}{\left(\frac{f(X_{i})}{p(\alpha ,X_{i})}\right)}^{2}$$

where $E_{p}\left[\xi \right]$ is the expectation of random variable ξ of pdf *p*. In this estimation, the ratios of the integrand and the pdf are squared causing high fluctuation and making the optimization process unstable.

Thus, instead of the variance, we need other optimization targets with more robust estimators in order to find the fractions $\alpha_{k}$ during adaptation.

## 5. MIS as Divergence between Distributions

MIS consists in looking for a distribution p(α,x) that mimics f(x) as much as possible. If we have non-negative integrand f(x)≥0 with integral μ=∫f(x)dx, then the integrand scaled down by the integral g(x)=f(x)/μ is also a distribution. The MIS problem can be stated as finding mixture pdf p(α,x) that minimizes the divergence between two distributions.

### 5.1. φ-Divergences

A φ-divergence $D_{\varphi }\left(q,r\right)$[20,21] is a measure of the dissimilarity between two probability distributions q(x) and r(x). It is defined by a convex function φ(t) satisfying φ(1)=0:

(11) $$D_{\varphi }\left(q,r\right)=\int r\left(x\right)\varphi \left(\frac{q(x)}{r(x)}\right)dx.$$

φ-divergences are always positive (by Jensen inequality) and are zero iff q≡r (for strict convexity) [21].

For instance, for φ(t)=tlogt, we obtain the Kullback–Leibler divergence:

(12) $$D_{\varphi }\left(q,r\right)=\int q\left(x\right)\log\frac{q(x)}{r(x)}dx=KL\left(q,r\right).$$

Note that φ-divergence is not symmetric, thus we have in principle two options to measure the dissimilarity of probability densities *q* and *r*, using either $D_{\varphi }\left(q,r\right)$ or $D_{\varphi }\left(r,q\right)$. Any of these two options can be used in practice, although they are not independent. If we define $\varphi^{*}\left(t\right)=t\varphi \left(\frac{1}{t}\right)$ then $D_{\varphi^{*}}\left(r,q\right)=D_{\varphi }\left(q,r\right)$[22]. For φ(t)=tlogt, we have $\varphi^{*}\left(t\right)=-\log t$.

The objective of importance sampling is to make probability density p(α,x) similar to scaled integrand g(x)=f(x)/μ, i.e., to minimize φ-divergence $D_{\varphi }\left(g,p\left(\alpha \right)\right)$. A φ-divergence is a convex function in both arguments and p(α) is a linear function of α, thus the functional is convex in α. This will ensure the existence of a minimum. With choosing $\varphi \left(t\right)=t^{2}-1$, we can get the variance back as a special case of this more general approach:

(13) $$V\left[F\right]=\int \frac{f^{2}\left(x\right)}{p(\alpha ,x)}dx-\mu^{2}=\mu^{2}\int p\left(\alpha ,x\right)\left({\left(\frac{g(x)}{p(\alpha ,x)}\right)}^{2}-1\right)dx=\mu^{2}D_{\varphi }\left(g\left(x\right),p\left(\alpha ,x\right)\right).$$

The optimal MIS problem is reduced to find the minimum of $D_{\varphi }\left(g,p\left(\alpha \right)\right)$ with the constraints $\sum_{i=1}^{m}\alpha_{i}=1$ and $\alpha_{i}\geq 0$. Using Lagrange multipliers, the solution is

(14) $$\frac{\partial D_{\varphi }\left(g,p\left(\alpha \right)\right)}{\partial \alpha_{i}}-\lambda =0,$$

which means that the derivatives of the divergence with respect to fractions $\alpha_{i}$ must be the same for each sampling method *i*.

The derivative can be expressed as

(15) $$\frac{\partial D_{\varphi }\left(g,p\left(\alpha \right)\right)}{\partial \alpha_{i}}=\int p_{i}\left(x\right)\left[\varphi \left(\frac{g(x)}{p(\alpha ,x)}\right)-\frac{g(x)}{p(\alpha ,x)}\varphi^{\prime }\left(\frac{g(x)}{p(\alpha ,x)}\right)\right]dx.$$

This integral is the expectation when samples are generated with pdf $p_{i}\left(x\right)$, thus we can write

(16) $$E_{p_{i}}\left[\varphi \left(\frac{g}{p(\alpha )}\right)-\frac{g}{p(\alpha )}\varphi^{\prime }\left(\frac{g}{p(\alpha )}\right)\right]=E_{p(\alpha )}\left[\varphi \left(\frac{g}{p(\alpha )}\right)-\frac{g}{p(\alpha )}\varphi^{\prime }\left(\frac{g}{p(\alpha )}\right)\right]=\lambda .$$

Equation (16) only holds when $\alpha_{i}>0$, i.e., in the interior of the simplex domain, not on the border.

Swapping the two distributions in the φ-divergence, we have another measure for the dissimilarity between p(α) and *g*, $D_{\varphi }\left(p\left(\alpha \right),g\right)$. Using Lagrange multipliers again, this leads to the following equation for unknown fractions $\alpha_{i}$

(17) $$\frac{\partial D_{\varphi }\left(p\left(\alpha \right),g\right)}{\partial \alpha_{i}}=\int p_{i}\varphi^{\prime }\left(\frac{p(\alpha )}{g}\right)dx=\lambda^{*}.$$

Using expected values, this option is expressed as

(18) $$E_{p_{i}}\left[\varphi^{\prime }\left(\frac{p(\alpha )}{g}\right)\right]=E_{p(\alpha )}\left[\varphi^{\prime }\left(\frac{p(\alpha )}{g}\right)\right]=\lambda$$

The problem with the optimization of the variance was that in the resulting equation the ratios of the sampled integrand and the pdf are squared (see Equation (10)), making the equation sensitive to non-optimal pdfs. With the proper definition of φ, we can solve this issue. For example, if φ(t)=−logt, then $\varphi^{\prime }=-1/t$, and the condition of optimality is

(19) $$E_{p_{i}}\left[\frac{g}{p(\alpha )}\right]=E_{p}\left[\frac{g}{p(\alpha )}\right]=-\lambda^{*}.$$

Remembering that g(x)=f(x)/μ, the last equation can also be written as

(20) $$E_{p_{i}}\left[\frac{f}{p(\alpha )}\right]=E_{p}\left[\frac{f}{p(\alpha )}\right],$$

which contains the ratios without squaring. This gives back the solution in [23], which is also conjectured in [16]. To further exploit this idea, we take the parametric family of Tsallis divergences. With its parameter the compromise between the robustness of the optimization equation and the similarity to the variance can be controlled.

### 5.2. Tsallis Divergences

Tsallis divergence [24] is associated with the Tsallis entropy [25]. Let us consider the one-parameter Tsallis divergence between distributions q(x) and r(x):

(21) $$\begin{array}{ccc} D_{\gamma }^{T}\left(q,r\right) & = & \frac{1}{\gamma -1}\left(\int \frac{q^{\gamma }\left(x\right)}{r^{\gamma -1}\left(x\right)}dx-1\right)\\ & = & \frac{1}{\gamma -1}\int \left(\frac{q^{\gamma }\left(x\right)}{r^{\gamma -1}\left(x\right)}-r\left(x\right)\right)dx=\frac{1}{\gamma -1}\int r\left(x\right)\left(\frac{q^{\gamma }\left(x\right)}{r^{\gamma }\left(x\right)}-1\right)dx. \end{array}$$

Tsallis divergence $D_{\gamma }^{T}\left(q,r\right)$ is a φ-divergence defined by

$$\varphi \left(t\right)=\frac{t^{\gamma }-1}{\gamma -1}.$$

Parameter γ should satisfy γ>0 and γ≠1, which guarantees that φ(t) exists and is convex.

It can be shown that $\lim_{\gamma \to 1}D_{\gamma }^{T}\left(q,r\right)=KL\left(q,r\right)$ is the Kullbach–Leibler divergence. When γ=2, Tsallis divergence turns to be the chi-square divergence:

(22) $$\begin{array}{ccc} \chi^{2}\left(q,r\right) & = & \int \frac{{\left(q\left(x\right)-r\left(x\right)\right)}^{2}}{r(x)}dx \end{array}$$

(23) $$\begin{array}{cc} & =\int \frac{q^{2}\left(x\right)}{r(x)}dx-2\int q\left(x\right)dx+\int r\left(x\right)dx\\ & =\int \frac{q^{2}\left(x\right)}{r(x)}dx-1=D_{2}^{T}\left(q,r\right). \end{array}$$

For γ=1/2, Tsallis divergence $D_{1/2}^{T}\left(q,r\right)$ becomes the squared Hellinger distance $H^{2}\left(q,r\right)$:

(24) $$\begin{array}{ccc} D_{1/2}^{T}\left(q,r\right) & = & -2\int (q{\left(x\right)}^{1/2}r{\left(x\right)}^{1/2}-q\left(x\right))dx\\ & = & 2\left(1-\int q{\left(x\right)}^{1/2}r{\left(x\right)}^{1/2}dx\right)=2H^{2}\left(q,r\right). \end{array}$$

Note that H(q,r) is a true metric.

### 5.3. Optimal MIS Solution with Tsallis Divergence

Optimal MIS needs to find fractions $\alpha_{i}$ that minimize $D_{\gamma }^{T}\left(g(x),p\left(\alpha ,x\right)\right)$ with the constraint $\sum_{i=1}^{m}\alpha_{i}=1$. Substituting function φ(t) of Tsallis divergence into Equation (16), we obtain that for all *i*, the quantities, called *γ-moments*,

(25) $$\begin{array}{c} M_{\gamma ,i}=\int {\left(\frac{f(x)}{p(\alpha ,x)}\right)}^{\gamma }p_{i}\left(x\right)dx=E_{p_{i}}\left[F^{\gamma }\right], \end{array}$$

have to be equal. Equation (25) guarantees a global minimum, as the function $D_{\gamma }^{T}\left(g\left(x\right),p\left(\alpha ,x\right)\right)$ is convex for all γ>0. Considering the case when g(x) and p(α,x) are swapped in the divergence, i.e., substituting function φ(t) of Tsallis into Equation (17), the requirement of the equality of the γ-moments is established again, but now γ should be replaced by 1−γ. Examining Equation (20) we can realize that optimizing for γ=1 is the same as minimizing the cross entropy or the Kullbach–Leibler divergence. Thus, the criterion of the equality of the γ-moments covers all cases.

### 5.4. Other φ-Divergences: Total Variation Divergence, $\chi_{P}^{k}$ and ${\left|\chi \right|}_{P}^{k}$ Divergences

The total variation can also be interpreted as a φ-divergence defined by $\varphi \left(t\right)=\frac{1}{2}\left|t-1\right|$:

$$TV\left(q,r\right)=\frac{1}{2}\int \left|q\left(x\right)-r\left(x\right)\right|dx$$

This is the only φ-divergence that is a true metric [26]. The condition of optimality is

(26) $$\int \mathrm{sign}\left(g\left(x\right)-p\left(\alpha ,x\right)\right)p_{i}\left(x\right)dx=\int \mathrm{sign}\left(g\left(x\right)-p\left(\alpha ,x\right)\right)p\left(\alpha ,x\right)dx$$

for all *i*, although we can hardly use Equation (26) to approximate a solution.

φ-divergences also include the Pearson–Vajda $\chi_{P}^{k}$ and ${\left|\chi \right|}_{P}^{k}$ divergences:

$$\chi_{P}^{k}\left(q,r\right)=\int \frac{{\left(q\left(x\right)-r\left(x\right)\right)}^{k}}{q^{k-1}\left(x\right)}dx,\;\;\;\;{\left|\chi \right|}_{P}^{k}\left(q,r\right)=\int \frac{{\left|q\left(x\right)-r\left(x\right)\right|}^{k}}{q^{k-1}\left(x\right)}dx$$

defined by the functions $\varphi \left(t\right)={\left(t-1\right)}^{k}$ and $\varphi \left(t\right)={\left|t-1\right|}^{k}$, respectively [17]. For k=2, we have $\chi_{P}^{2}\left(q,r\right)={\left|\chi \right|}_{P}^{2}\left(q,r\right)=\chi^{2}\left(r,q\right)$, and for k=1, identities $\chi_{P}^{1}\left(q,r\right)=0$ and ${\left|\chi \right|}_{P}^{1}\left(q,r\right)=2TV\left(q,r\right)$ hold.

## 6. Finding the Optimal Solution

For the sake of simplicity, we assume that two sampling methods of pdfs $p_{1}\left(x\right)$ and $p_{2}\left(x\right)$, associated with fractions α and 1−α are combined. The mixture density is

$$p\left(\alpha ,x\right)=\alpha p_{1}\left(x\right)+\left(1-\alpha \right)p_{2}\left(x\right).$$

From the optimality condition of Equation (25), we obtain

(27) $$\int {\left(\frac{f(x)}{p(\alpha ,x)}\right)}^{\gamma }\left(p_{1}\left(x\right)-p_{2}\left(x\right)\right)dx=\zeta \left(\alpha \right)=0.$$

This is a non-linear equation for unknown fraction α, which is solved with Newton–Raphson iteration. The derivative of ζ(α) is

(28) $$\zeta^{\prime }\left(\alpha \right)=\frac{\partial \zeta (\alpha )}{\partial \alpha }=-\gamma \int \frac{f^{\gamma }\left(x\right)}{p^{\gamma +1}\left(\alpha ,x\right)}{\left(p_{1}\left(x\right)-p_{2}\left(x\right)\right)}^{2}dx,$$

as $\partial p(\alpha ,x)/\partial \alpha =p_{1}\left(x\right)-p_{2}\left(x\right)$.

The value of ζ(α) and its derivative can be estimated in the following way. Let us re-write ζ(α) as an expectation:

(29) $$\zeta \left(\alpha \right)=\int \frac{f^{\gamma }\left(x\right)}{p^{\gamma +1}\left(\alpha ,x\right)}\left(p_{1}\left(x\right)-p_{2}\left(x\right)\right)p\left(\alpha ,x\right)dx=E_{p(\alpha )}\left[\frac{f^{\gamma }\left(X\right)}{p^{\gamma +1}\left(\alpha ,X\right)}\left(p_{1}\left(X\right)-p_{2}\left(X\right)\right)\right].$$

Taking *N* samples $\left\{X_{1},X_{2},\ldots ,X_{N}\right\}$ according to pdf p(α,x), we have the estimator

(30) $$\hat{\zeta (\alpha )}=\frac{1}{N}\sum_{i=1}^{N}\frac{f^{\gamma }\left(X_{i}\right)}{p^{\gamma +1}\left(\alpha ,X_{i}\right)}\left(p_{1}\left(X_{i}\right)-p_{2}\left(X_{i}\right)\right),$$

and in the same way,

(31) $$\hat{\zeta^{\prime }\left(\alpha \right)}=-\frac{\gamma }{N}\sum_{i=1}^{N}\frac{f^{\gamma }\left(X_{i}\right)}{p^{\gamma +2}\left(\alpha ,X_{i}\right)}{\left(p_{1}\left(X_{i}\right)-p_{2}\left(X_{i}\right)\right)}^{2}.$$

The integrals are estimated with an iterative algorithm updating α after each iteration. Starting with $\alpha^{\left(0\right)}=1/2$, in iteration *k* we draw *N* samples according to $p(\alpha^{\left(k-1\right)},x)$, compute $\hat{\zeta (\alpha^{\left(k-1\right)})}$ and $\hat{\zeta^{\prime }\left(\alpha^{\left(k-1\right)}\right)}$, and use the Newton–Raphson formula to obtain updated fraction $\alpha^{\left(k\right)}$:

(32) $$\alpha^{\left(k\right)}=\alpha^{\left(k-1\right)}-\frac{\hat{\zeta (\alpha^{\left(k-1\right)})}}{\hat{\zeta^{\prime }\left(\alpha^{\left(k-1\right)}\right)}}.$$

## 7. Numerical Examples

We present here three 1D examples. We used the Mathematica package for the computations.

### 7.1. Example 1

**Figure 1:**
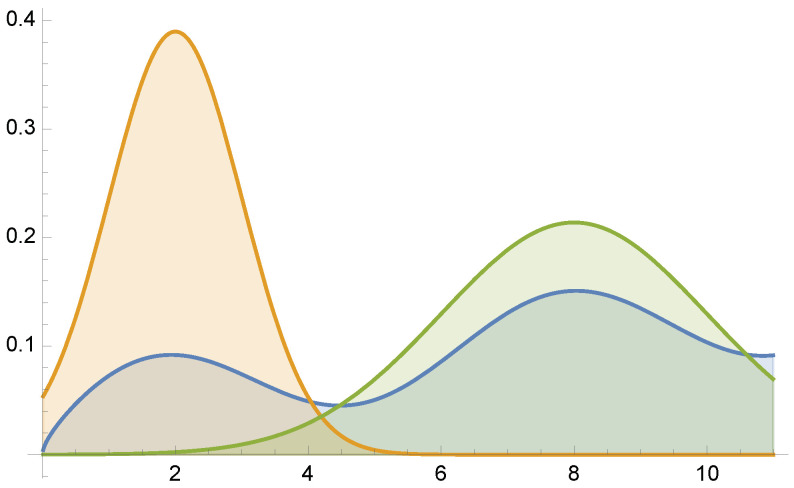
Example 1: f(x)/μ (in blue) superimposed on the two pdfs used for MIS integration.

Suppose we want to evaluate the integral (see Figure 1)

(33) $$\mu =\int_{0.01}^{3.5\pi }\left(\sqrt{x}+\sin x\right)dx\approx 25.3065$$

by MIS combining sampling methods of pdfs Gauss(2,1) and Gauss(8,2), where Gauss(m,σ) stands for the normal distribution of mean *m* and standard deviation σ. For this example, equal sample number MIS has variance V[F]=24.1152, and the minimum variance is V[F]=13.4788.

**Figure 2:**
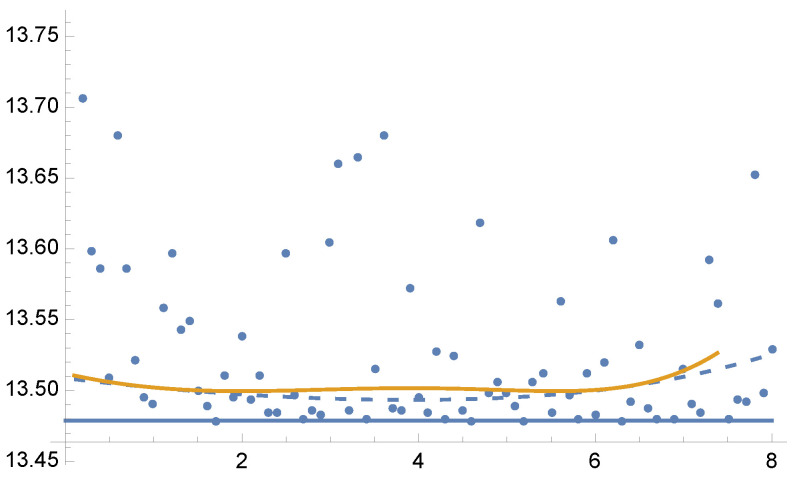
Example 1: Values of V[F] (dashed line) and V[F] (continuous line) for the solution of Equation (25) for each value of parameter γ from 0.1 to 8, in the horizontal axis. Variance V[F] (continuous line) is minimal when γ=2, as V[F] corresponds to $\chi^{2}$ divergence (Equation (13)). Dots show the variance V[F] for the optimal α values for each parameter γ using 5 Newton–Raphson iterations with 100 total samples in each iteration. Horizontal line corresponds to the minimum variance V[F]=13.4788. A zoom-out is shown in Figure 3.

In Figure 2, we show the variances V[F] and V[F] for the optimal solution of Tsallis divergences from γ=0.1 to γ=8 in steps of 0.1. Dots depict the values of V[F] for the optimal α fractions for each parameter γ using 5 Newton–Raphson iterations with 100 total samples in each iteration.

**Figure 3:**
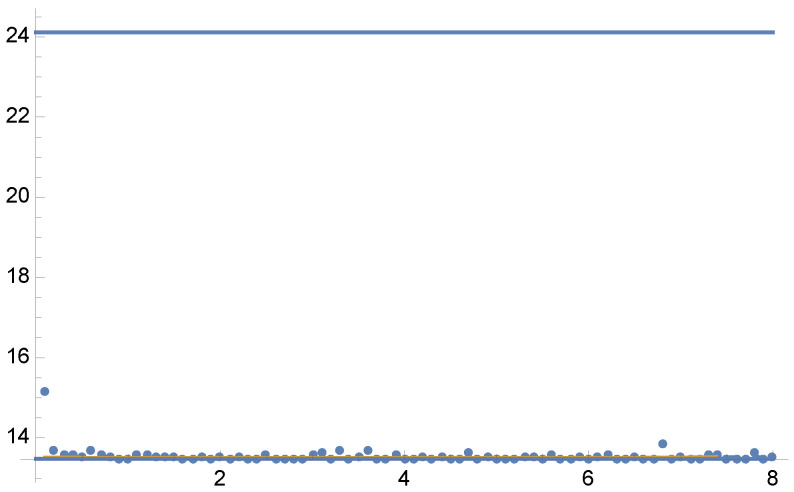
Example 1. Zoom-out of Figure 2. Observe that V[F]<V[F] except for γ=1 where V[F]=V[F]. For the equal sample budget case, V[F]=24.1152, while the minimum is V[F]=13.4788.

### 7.2. Example 2

**Figure 4:**
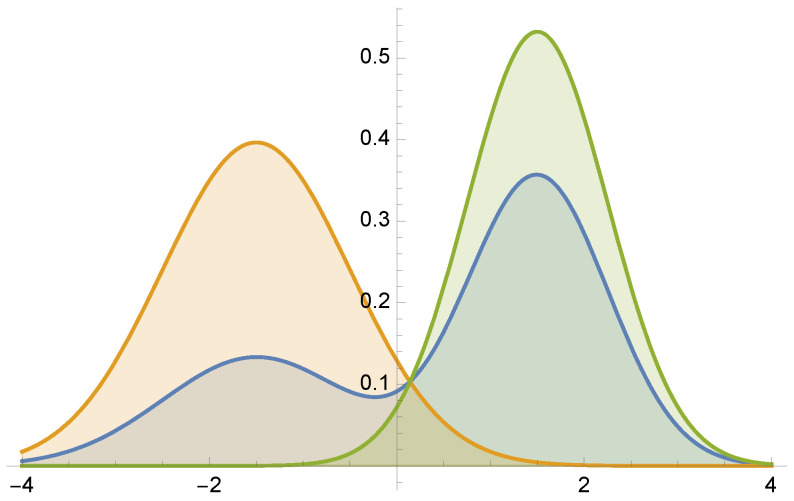
Example 2: f(x)/μ (in blue) superimposed on the two pdfs used for MIS integration.

**Figure 5:**
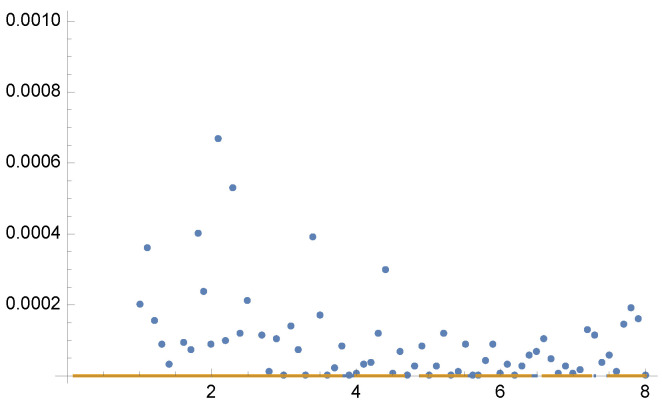
Example 2: V[F] values corresponding to the Newton–Raphson solutions for the optimal α values (vertical axis), from γ=0.1 to γ=8 in steps of 0.1 (horizontal axis) for each parameter γ. Dots show the variance V[F] for the optimal α values for each parameter γ using 5 Newton–Raphson iterations with 100 total samples in each iteration. Minimum variance is V[F]=0. A zoom-out is shown in Figure 6.

**Figure 6:**
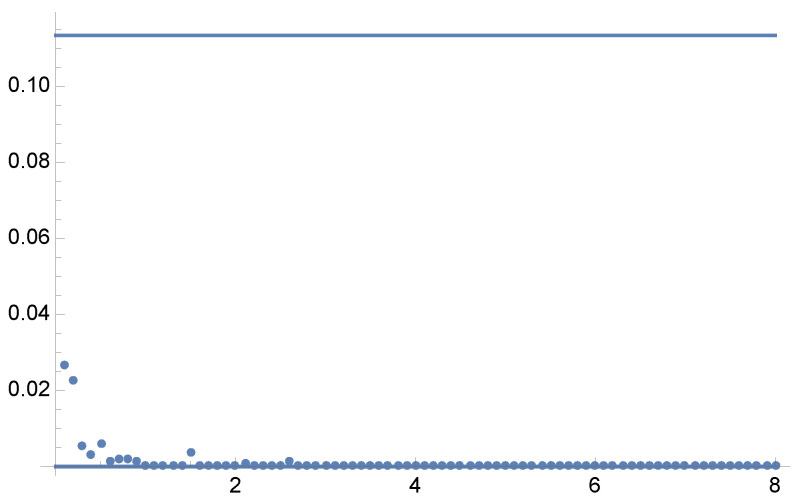
Example 2: Zoom-out of Figure 5. The minimum of V[F] and V[F] is 0. For equal sample budget MIS, the variance is V[F]=0.1134.

Let us consider integral (see Figure 4)

(34) $$\mu =\int_{-4}^{4}Gauss\left(-1.5,1\right)+2Gauss\left(1.5,0.75\right)dx\approx 2.9929$$

by MIS using functions Gauss(−1.5,1) and Gauss(1.5,0.75). For this example, equal sample number MIS has a variance of V[F]=0.1134, and the minimum variance is V[F]=0. Figure 5 and Figure 6 present the optimal solutions and the results after 5 Newton–Raphson iterations with 100 total samples in each iteration.

### 7.3. Example 3

**Figure 7:**
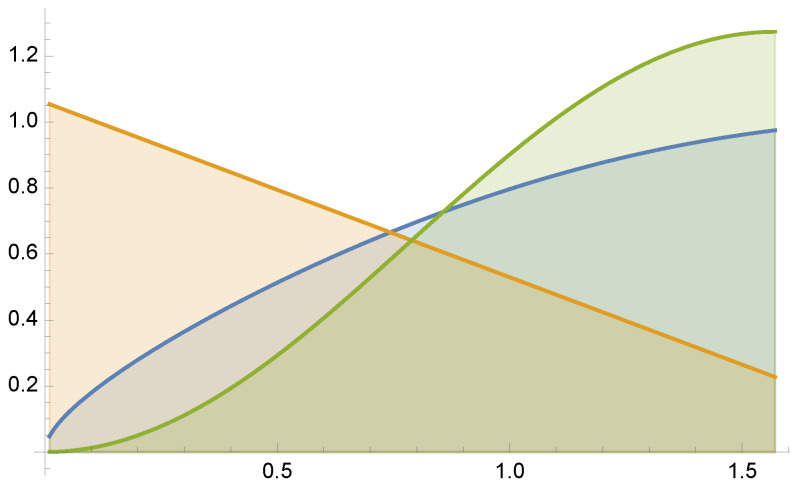
Example 3: f(x)/μ (in blue) superimposed on the two pdfs used for MIS integration.

**Figure 8:**
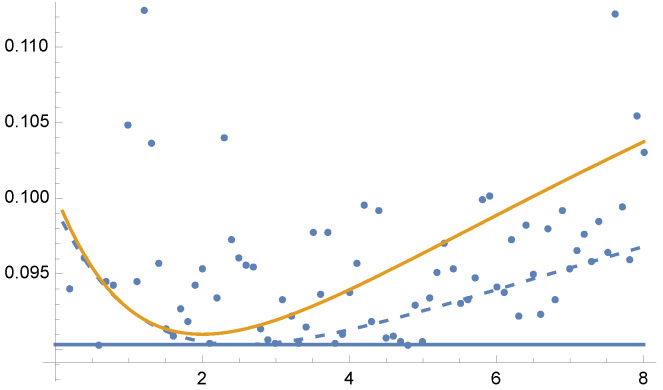
Example 3: V[F] (dashed line) and V[F] (continuous line), for the solution of Equation (25) for each value of the parameter γ between 0.1 and 8, in the horizontal axis. Variance V[F] (continuous line) is minimal when γ=2, as V[F] corresponds to $\chi^{2}$ divergence (Equation (13)). Dots are the V[F] values after 5 Newton–Raphson iterations taking 100 samples in total at each iteration. Horizontal line corresponds to minimum variance, V[F]=0.09032. A zoom-out is shown in Figure 9.

**Figure 9:**
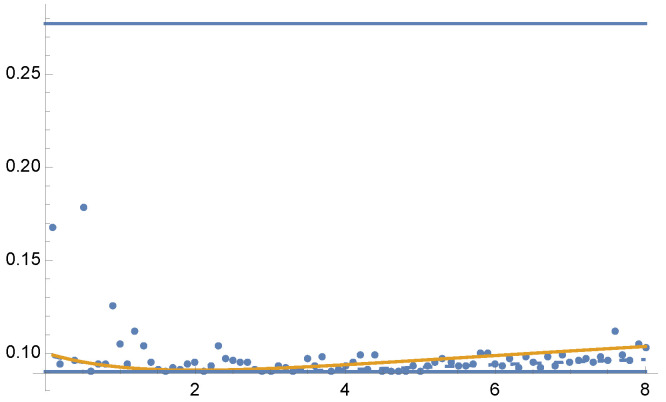
Example 3: Zoom-out of Figure 8. For equal sample budget V[F]=0.2772, and minimum is V[F]=0.09032.

Finally, consider the approximation of the following integral (see Figure 7)

(35) $$\begin{array}{c} \;\mu =\int_{0.01}^{\pi /2}\left(\sqrt{x}+\sin x\right)dx\approx 2.3118. \end{array}$$

by MIS using functions 2−x, and $\sin^{2}\left(x\right)$. For this example, equal sample budget MIS has a variance of V[F]=0.2772, and the minimum variance is V[F]=0.09032. In Figure 8 and Figure 9 we show the variances V[F] and V[F] for the optimal solution for parameters from γ=0.1 to γ=8. Dots are the results of V[F] using 5 Newton–Raphson iterations with 100 total samples in each iteration.

## 8. Combination of Light Source Sampling and BRDF Sampling in Computer Graphics

Here we consider a classical problem of computer graphics, the combination of light source sampling and BRDF (Bidirectional Reflectance Distribution Function) sampling. To compute the reflected radiance $L^{r}\left(\vec{p},\vec{\omega }\right)$ of a surface point $\vec{p}$ at direction $\vec{\omega }$, the following integral should be evaluated in the hemispherical domain of incident directions Ω:

(36) $$L^{r}\left(\vec{p},\vec{\omega }\right)=\int_{\Omega }L\left({\vec{p}}^{\prime },{\vec{\omega }}^{\prime }\right)f_{r}\left({\vec{\omega }}^{\prime },\vec{p},\vec{\omega }\right)\cos\theta^{\prime }d\omega^{\prime }$$

where $L({\vec{p}}^{\prime },{\vec{\omega }}^{\prime })$ is the radiance of point ${\vec{p}}^{\prime }$ visible from point $\vec{p}$ in incident direction $-{\vec{\omega }}^{\prime }$, $f_{r}\left({\vec{\omega }}^{\prime },\vec{p},\vec{\omega }\right)$ is the BRDF expressing the portion of the light beam that is reflected from direction ${\vec{\omega }}^{\prime }$ to $\vec{\omega }$ at point $\vec{p}$ and $\theta^{\prime }$ is the angle between the surface normal at point $\vec{p}$ and incident direction $-{\vec{\omega }}^{\prime }$. While solving such problems, we have two sampling methods, $p_{1}\left({\vec{\omega }}^{\prime }\right)$ approximately mimicking the $f_{r}\left(\vec{\omega },\vec{p},{\vec{\omega }}^{\prime }\right)\cos\theta^{\prime }$ factor and $p_{2}\left({\vec{\omega }}^{\prime }\right)$ mimicking the incident radiance $L({\vec{p}}^{\prime },{\vec{\omega }}^{\prime })$.

MIS would use a combined pdf in the following form

(37) $$p\left(\alpha ,{\vec{\omega }}^{\prime }\right)=\alpha p_{1}\left({\vec{\omega }}^{\prime }\right)+\left(1-\alpha \right)p_{2}\left({\vec{\omega }}^{\prime }\right).$$

where the task is the optimal determination of weight α.

In order to test the proposed method, we render the classic scene of Veach [1] with combined light source and BRDF sampling. The shiny rectangles have max-Phong BRDF [27] with shininess parameters 50, 100, 500, and 1000, respectively. The four spherical light sources emit the same power.

**Figure 10:**
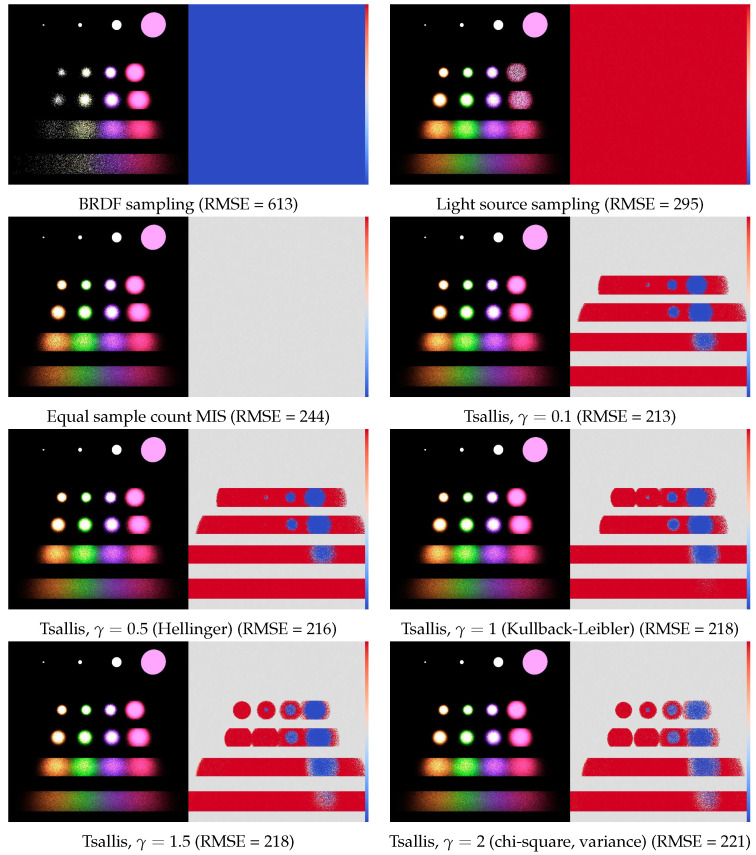
Comparison of MIS weighting schemes for the direct lighting problem of computer graphics. The left part is the image rendered with 100 rays per pixel, the right part is weight α of the light source sampling. The RMSE is computed as the average of 30 independent executions. The (0,1) interval of possible α values is visualized by the color bar.

**Figure 11:**
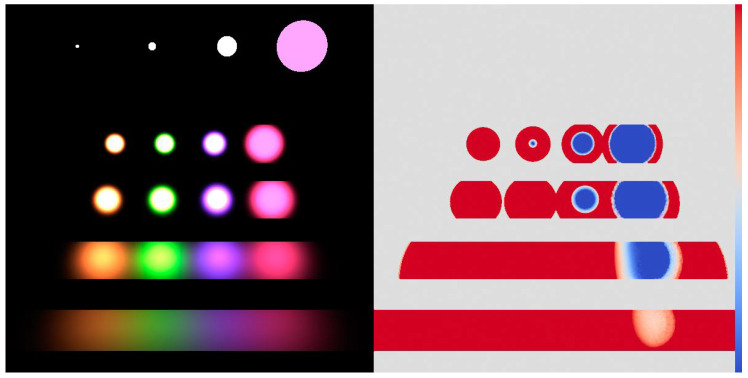
Reference image and α-map obtained with 50,000 samples per pixel organized in 5 iterations.

**Figure 12:**
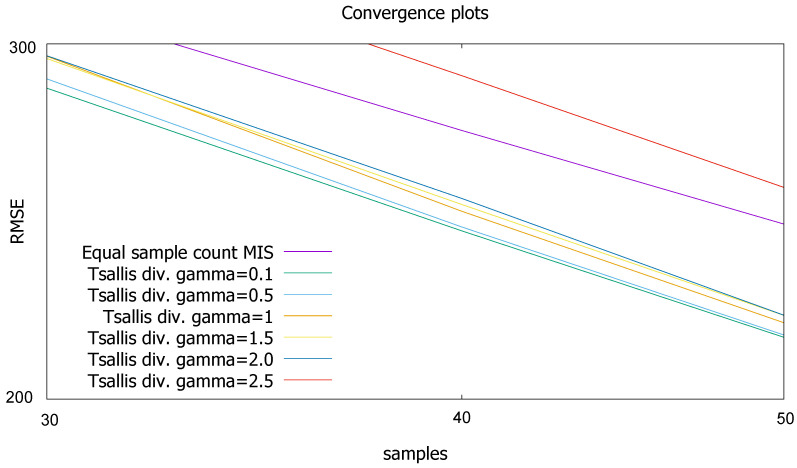
RMSE as functions of the number of samples per pixel.

For each pixel, we used 50 samples in total organized in 5 iterations. The process starts with 5 BRDF and 5 light source samples per pixel, and the per-pixel α weights are updated at the end of each iteration. Figure 10 shows the rendered images together with the α-maps, and we compare the original sampling techniques, equal count MIS, variance minimization, and Tsallis divergence. The reference obtained by high number of samples is shown by Figure 11. Table 1 depicts the Root Mean Square Error (RMSE) values. Figure 12 shows the convergence plots.

Finally, we note that φ-divergences were used for global illumination as oracles for adaptive sampling in [28].

## 9. Discussion

The goal of adaptive MIS is to find the weights of given pdfs that eventually lead to minimal integration error. The definition of the error may be application dependent, in this paper we chose the RMSE, which describes the variance of the estimator. We argued that instead of the direct optimization of the estimated variance, it is better to use other measures for the optimization target since the variance estimation may strongly fluctuate if the sample number is moderate, which prevents the adaptation process from converging to the real optimum. The alternative criterion is the Tsallis divergence, which includes important particular divergence types and by setting its parameter, more robust targets can be defined.

In order to demonstrate the proposed method, we took three numerical examples and a fundamental problem of computer graphics. We can observe in all examples that the adaptive MIS can significantly outperform the equal sample count MIS, thus examining adaptation strategies has theoretical and practical relevance. In the first numerical example, none of the combinations of the two sampling pdfs could well mimic the integrand, thus the computation errors are quite high and the influence of parameter γ is small. In the second numerical example, the integrand is proportional to an appropriate linear combination of the two sampling pdfs, thus zero variance estimator is possible. If parameter γ is not too small, the method could indeed compute the integral with high accuracy. This means that in easy integration problems, Tsallis divergence can be safely used. The third numerical example is a more difficult, realistic case when sufficiently high number of samples are used in each iteration step to estimate the optimization target. As expected, the optimal value of parameter γ is close to 2 since this is the point where Tsallis divergence becomes the variance V[F].

In the image synthesis example, the integrand is not a weighted sum but a product where the sampling pdfs approximately mimic the factors. In this case, no zero variance MIS is possible. Unlike in the numerical examples, we took small number of samples for each pixel according to practical scenarios. The small sample number made the optimization target equation unreliable unless parameter γ is reduced from 2 that corresponds to the variance. Looking at Figure 10, we can observe that when γ becomes smaller, the computed weight maps become slightly less accurate in average but also less noisy, which eventually reduces the computation error.

We have also seen in the examples that the differences between the variances corresponding to the optimal values for the different parameters γ of the Tsallis divergence are small. This is in accordance with any φ-divergence being a second order approximation of $\chi^{2}$ divergence [17], thus obtaining any minimum within a wide range of γ parameters would be a good solution. Aside from γ=2, which corresponds to V[F], the most interesting Tsallis divergence from a practical perspective is the Kullback-Leibler divergence, corresponding to γ=1.

## 10. Conclusions and Future Work

We have shown in this paper that finding the optimal weights for MIS can be presented as finding the minimum φ-divergence between the normalized integrand and the importance pdf. Being convex, a φ-divergence allows convex optimization. We have singled out the Tsallis divergences with parameter γ as they have the further advantage that the equations for the optimal value consist of making the γ-moments equal. We have tested our results with numerical examples and solving an illumination problem.

In our future work we will investigate the optimal efficiencies, i.e., taking into account the cost of sampling. This has been done for the variance V[F] (i.e., γ=2) in [4,6] where the implicit equation for the optimal solution was given. However, the efficiency equation does not allow convex optimization. Using the γ-moments, or a different family of φ-divergences, could help in obtaining a robust solution for the efficiency.

## Figures and Tables

**Table 1:** RMSE of different MIS methods.

Method	RMSE
BRDF sampling	613
Light source sampling	295
Equal count MIS	244
Tsallis (γ=0.01)	216
Tsallis (γ=0.1)	213
Tsallis (γ=0.5) (Hellinger)	215
Tsallis (γ=1) (Kullback-Leibler)	218
Tsallis (γ=1.5)	218
Tsallis (γ=2) (chi-square, variance)	221
Tsallis (γ=2.5)	254

## Data Availability

Not applicable.
